# From rhythm to resilience: The interplay of circadian rhythm, melatonin, lifestyle, nutrition in psychoneuroimmunology (PNI)

**DOI:** 10.37796/2211-8039.1691

**Published:** 2025-12-01

**Authors:** Chang Jane Pei-Chen, Su Kuan-Pin

**Affiliations:** a Mind-Body Interface Research Center (MBI Lab & Care), China Medical University Hospital, Taichung, Taiwan; b Child Psychiatry Division, Department of Psychiatry, China Medical University Hospital, Taichung, Taiwan; c College of Medicine, China Medical University (CMU), Taichung, Taiwan; d An-Nan Hospital, China Medical University (CMU), Tainan, Taiwan; e Office of Research and Development, Asia University, Taichung, Taiwan

**Keywords:** ADHD, Cardiovascular diseases, Circadian rhythm, Lifestyle, MDD, Nutrition, Psychoneuroimmunology (PNI)

## Abstract

Emerging evidence reveals that circadian rhythm, melatonin signaling, nutrition, and inflammation are intricately intertwined in shaping both mental and physical health. Circadian disruptions and lifestyle imbalances contribute to neuroinflammation, mood dysregulation, and cardiometabolic dysfunction, as seen in attention-deficit/hyperactivity disorder (ADHD), major depressive disorder (MDD), metabolic syndrome, and cardiovascular diseases. This special issue highlights interdisciplinary research that integrates circadian biology, nutrition, and psychoneuroimmunology (PNI) toward personalized and preventive psychiatry. Featured studies explore circadian and melatonin mechanisms in ADHD and MDD, the therapeutic and prophylactic potential of omega-3 polyunsaturated fatty acids (n-3 PUFAs), the neuroprotective potentical of paeoniflorin, and the role of extracellular matrix gene variants in MDD vulnerability. Additionally, evidence supporting lifestyle-based interventions such as Tai Chi underscores the value of non-pharmacological mind–body approaches. Collectively, these studies illuminate a multidimensional model linking biological rhythm, nutrition, and inflammation to mental resilience and cardiovascular well-being.

## 1. Introduction

The interplay among circadian rhythms, nutrition, and lifestyle provides new perspectives for addressing common mental and physical disorders. The body's internal clock orchestrates circadian rhythms, playing a crucial role in regulating immune function and neurochemical homeostasis [1]. Disruptions in these rhythms have been implicated in the development of various neuropsychiatric conditions, such as attention-deficit hyperactivity disorder (ADHD) [2] and major depressive disorder (MDD) [3], as well as in cardiovascular diseases [4]. Furthermore, growing evidence highlights inflammation as a shared underlying mechanism among these disorders. Elevated inflammatory markers have been observed in ADHD [5], MDD [6], and cardiovascular diseases, particularly when comorbid with MDD [7]. In contrast, healthy nutritional habits and lifestyle interventions—such as regular physical activity and improved sleep hygiene—have shown promise in reducing neuroinflammation, restoring circadian balance, and promoting both mental and cardiovascular health [8].

## 2. Off the beat with circadian rhythm and singing the blues with a broken heart

Disruptions in circadian rhythms and melatonin secretion have been closely linked with a range of neuropsychiatric disorders—including ADHD [2,9], Alzheimer's disease [10], anxiety and depression [3,11], bipolar disorder [12], suicide attempts [13], as well as physical conditions such as coronary artery disease [4], irritable bowel syndrome [14], migraine [10], and pain [15]. Inflammation appears to be the critical link connecting circadian and melatonin dysregulation to these disorders. For instance, elevated inflammatory biomarkers, including interleukin-6 (IL-6) and C-reactive protein (CRP), are observed in ADHD [5] and MDD [6,16]. Genetic polymorphisms in proinflammatory genes, such as *IL-6* (−174G > C) and *TNF-α* (−308G > A), have also been associated with heightened susceptibility to acute coronary syndromes [17]. On the other hand, melatonin exhibits notable anti-inflammatory properties through its capacity to scavenge reactive oxygen species and mitigate oxidative damage across multiple organ systems [1]. It further inhibits the nuclear translocation of nuclear factor-kappa B (NF-κB) and its subsequent DNA binding, thereby downregulating proinflammatory cytokines such as interleukins and tumor necrosis factor-alpha [1].

## 3. Fine tuning with nutrition, melatonin, and behavioral intervention

Emerging research in Lifestyle Medicine underscores the therapeutic potential of nutritional supplementation and physical activity in mitigating symptoms of ADHD, MDD, and cardiovascular disease. Nutritional interventions, particularly omega-3 polyunsaturated fatty acids (n-3 PUFAs), have demonstrated beneficial effects in alleviating clinical symptoms of ADHD [18] and MDD [19–21]. Iqbal et al. suggested that n-3 PUFAs may exert these effects by counteracting melatonin and circadian dysregulation in ADHD [2], while Malau et al. highlighted their prophylactic role in MDD [22]. Zailani et al. further explored the genetic variations in extracellular matrix degradation pathways that may contribute to MDD pathophysiology [23]. Additional compounds, such as paeoniflorin, have also been proposed to possess antidepressant potential [24]. Le et al. reinforced the role of melatonin in neuropsychiatric disorders through evidence spanning preclinical and clinical studies [25]. Beyond nutrition, physical activity is pivotal. Cheng et al. demonstrated that Tai Chi enhances cardiovascular fitness, lowers blood pressure, improves vascular function, and simultaneously reduces psychological stress while fostering emotional balance [26].

## 4. Personalized treatment with multidisciplinary approach and keeping rhythm

In an era of chronic stress and digital stimulation, maintaining internal balance is vital. Lifestyle Medicine—including circadian alignment, targeted nutrition, and regular movement—must be emphasized as a preventive strategy. Advancements in molecular research pave the way for genetically based personalized interventions. Ultimately, harmonizing the ‘inner orchestra’ is the holistic strategy required to mitigate inflammation and reduce the global burden of mind–body disorders [8].
